# Bis{tris­[3-(2-pyrid­yl)-1*H*-pyrazole]iron(II)} dodeca­molybdo(V,VI)phosphate hexa­hydrate

**DOI:** 10.1107/S1600536810004861

**Published:** 2010-02-13

**Authors:** Lujiang Hao, Tongjun Liu, Jiangkui Chen, Xiaofei Zhang

**Affiliations:** a College of Food and Biological Engineering, Shandong Institute of Light Industry, Jinan 250353, People’s Republic of China

## Abstract

Crystals of the title compound, [Fe(C_8_H_7_N_3_)_3_]_2_[PMo_12_O_40_]·6H_2_O, prepared under hydro­thermal conditions, are isotypic with the Mn^2+^ and Cd^2+^ analogues. The Fe^2+^ cation is in a distorted octa­hedral coordination by six N atoms from three chelating 3-(2-pyrid­yl)-1*H*-pyrazole ligands. The heteropoly­anion [PMo_12_O_40_]^4−^ is a one-electron reduced species in which two O atoms of the central PO_4_ group (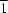 symmetry) are equally disordered about an inversion centre. N—H⋯O and O—H⋯O hydrogen bonds make a contribution to the crystal packing. The Fe—N bond lengths [2.085 (19)—2.15 (2) Å] are somewhat shorter than the Mn—N and Cd—N bond lengths [2.224 (6)–2.283 (5) and 2.316 (7)–2.334 (6) Å, respectively]. All other bond lengths and angles and the hydrogen-bonding motifs are very similar in the isotypic structures.

## Related literature

For the isotypic Mn^2+^ and Cd^2+ ^structures, see: Hao, Ma *et al.* (2010 ▶); Hao, Wang *et al.* (2010 ▶). For general background to polyoxometalates, see: Pope & Müller (1991 ▶). For polyoxometalates modified with amines, see: Zhang, Dou *et al.* (2009 ▶); Zhang, Wei *et al.* (2009 ▶). For other structures containing the one-electron reduced heteropolyanion [PMo_12_O_40_]^4−^, see: Artero & Proust (2000 ▶); Kurmoo *et al.* (1998 ▶); Niu *et al.* (1999 ▶). For the role of amines in hydro­thermal synthesis, see: Yang *et al.* (2003 ▶).
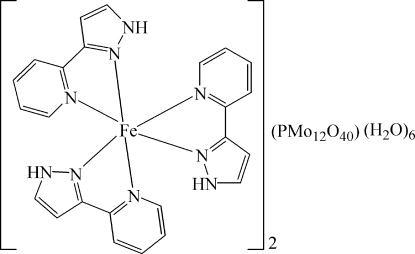


## Experimental

### 

#### Crystal data

[Fe(C_8_H_7_N_3_)_3_]_2_[PMo_12_O_40_]·6H_2_O
*M* *_r_* = 2913.04
Monoclinic, 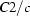

*a* = 18.6816 (10) Å
*b* = 16.2224 (10) Å
*c* = 27.6405 (15) Å
β = 104.295 (1)°
*V* = 8117.4 (8) Å^3^
*Z* = 4
Mo *K*α radiation
μ = 2.26 mm^−1^
*T* = 293 K
0.12 × 0.10 × 0.08 mm

#### Data collection

Bruker APEXII CCD diffractometer
Absorption correction: multi-scan (*SADABS*; Bruker, 2001 ▶) *T*_min_ = 0.773, *T*_max_ = 0.840
20565 measured reflections
7090 independent reflections
5550 reflections with *I* > 2σ(*I*)
*R*_int_ = 0.042

#### Refinement

*R*[*F* ^2^ > 2σ(*F* ^2^)] = 0.039
*wR*(*F* ^2^) = 0.142
*S* = 1.00
7090 reflections
592 parameters
H-atom parameters constrained
Δρ_max_ = 1.36 e Å^−3^
Δρ_min_ = −0.80 e Å^−3^

### 

Data collection: *APEX2* (Bruker, 2004 ▶); cell refinement: *SAINT-Plus* (Bruker, 2001 ▶); data reduction: *SAINT-Plus*; program(s) used to solve structure: *SHELXS97* (Sheldrick, 2008 ▶); program(s) used to refine structure: *SHELXL97* (Sheldrick, 2008 ▶); molecular graphics: *SHELXTL* (Sheldrick, 2008 ▶); software used to prepare material for publication: *SHELXTL*.

## Supplementary Material

Crystal structure: contains datablocks global, I. DOI: 10.1107/S1600536810004861/wm2304sup1.cif

Structure factors: contains datablocks I. DOI: 10.1107/S1600536810004861/wm2304Isup2.hkl

Additional supplementary materials:  crystallographic information; 3D view; checkCIF report

## Figures and Tables

**Table 1:** Selected bond lengths (Å)

Fe1—N8	2.085 (19)
Fe1—N5	2.08 (2)
Fe1—N2	2.11 (2)
Fe1—N1	2.129 (17)
Fe1—N4	2.13 (2)
Fe1—N7	2.15 (2)
P1—O21*A*^i^	1.49 (2)
P1—O21*B*^i^	1.52 (2)
P1—O19*B*^i^	1.54 (2)
P1—O19*A*^i^	1.56 (2)

Symmetry code: (i) 
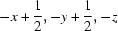
.

**Table 2:** Hydrogen-bond geometry (Å, °)

*D*—H⋯*A*	*D*—H	H⋯*A*	*D*⋯*A*	*D*—H⋯*A*
N3—H3*A*⋯O17^ii^	0.86	2.03	2.82 (3)	153
N6—H6⋯O2*W*	0.86	1.98	2.82 (5)	167
N9—H9*A*⋯O1*W*	0.86	1.95	2.77 (3)	157

Symmetry code: (ii) 
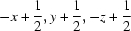
.

**Fig. 1.**
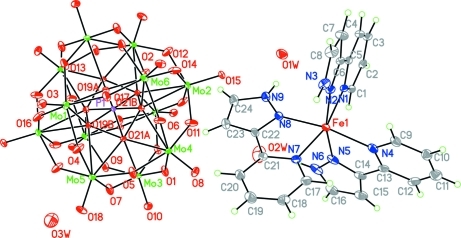
The building blocks of the title compound with the atom-labelling scheme. Displacement ellipsoids are drawn at the 30% probability level; H atoms are given as spheres of arbitrary radius.

**Fig. 2.**
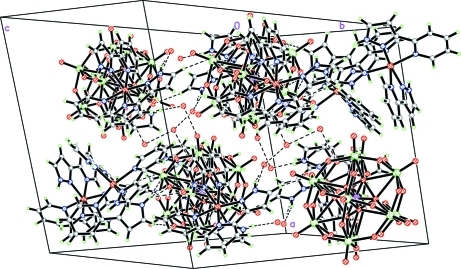
The crystal packing of the title compound, displayed with N—H···O and O—H···O hydrogen bonds as dashed lines.

## supplementary crystallographic information

### Comment

The design and synthesis of polyoxometalates has attracted continuous research interest not only because of their appealing structural and topological novelties, but also due to their interesting optical, electronic, magnetic, and catalytic properties, as well as their potential medical applications (Pope & Müller, 1991). In our research group, organic amines, such as 3-(2-pyridyl)-1*H*-pyrazole and pyrazine, are used to effectively modify polyoxomolybdates under hydrothermal condictions (Zhang, Dou *et al.*, 2009; Zhang, Wei *et al.*, 2009). Here, we describe the synthesis and structural characterization of the title compound.

As shown in Figure 1, the asymmetric unit of the title compound consists of three subunits, *viz.* of a complex [Fe(C_8_H_7_N_3_)_3_]^2+^ cation, half of a a [PMo_12_O_40_]^4-^ heteropolyanion and of three uncoordinated water molecules. The Fe^2+^ ion is in a distorted octahedral coordination by six N atoms from three chelating 3-(2-pyridyl)-1*H*-pyrazole ligands.

The heteropolyanion [PMo_12_O_40_]^4-^ anion is an one-electron reduced derivative of [PMo_12_O_40_]^3-^, similar to anions with different counter cations as reported by Artero & Proust (2000); Kurmoo *et al.* (1998); Niu *et al.* (1999). The employed organic ligand appears to adjust the pH value, and additionally supplies reducing electrons, which is a commonly observed feature of hydrothermal syntheses when organic amines are used to prepare various hybrid materials, zeolites or metal phosphates (Yang *et al.*, 2003).

In the Keggin-type heteropolyanion, each Mo atom is surrounded by six O atoms and the P atom is located at the center of the anion. There are four kinds of O atoms present in the anion according to their coordination environments: O*_a_* (O atoms in the disordered PO_4_ tetrahedron), O*_b_* (bridging O atoms between two triplet groups of MoO_6_ octahedra), O*_c_* (bridging O atoms within one triplet group of MoO_6_ octahedra) and O*_d_* (terminal O atoms). The P—O bond distances are in the normal range of 1.49 (2)—1.56 (2) Å. The Mo—O bond distances vary widely from 1.636 (15) to 2.51 (2) Å. The shortest Mo—O bonds are in the range of 1.636 (2)—1.659 (15) Å for the terminal oxygen atoms. The longest Mo—O lengths are in the range of 2.43 (2)—2.51 (2) Å for those oxygen atoms connected with both Mo and P atoms.

N—H···O and O—H···O hydrogen bonding between the cationic and anionic moieties and the uncoordinated water molecules leads to a consolidation of the structure (Fig. 2; Table 2).

The crystal structure of [(Fe(C_8_H_7_N_3_)_3_]_2_[PMo_12_O_40_](H_2_O)_6_ is isotypic with the Mn^2+^ and Cd^2+^ analogues, [(Mn(C_8_H_7_N_3_)_3_]_2_[PMo_12_O_40_](H_2_O)_6_ (Hao, Ma *et al.*, 2010) and [(Cd(C_8_H_7_N_3_)_3_]_2_[PMo_12_O_40_](H_2_O)_6_ (Hao, Wang *et al.*, 2010). In comparison with the Mn^2+^ and Cd^2+^ analogues, the Fe—N bond lengths (2.085 (19)—2.15 (2) Å) are somewhat shorter than the Mn—N and Cd—N bond lengths with 2.224 (6)—2.283 (5) Å and 2.316 (7)—2.334 (6) Å, respectively. All other bond lengths and angles and the hydrogen-bonding motifs are very similar in the isotypic structures.

### Experimental

A mixture of 3-(2-pyridyl)-1*H*-pyrazole (0.5 mmoL 0.07 g), sodium molybdate (0.4 mmoL, 0.10 g), iron(III) chloride hexahydrate (0.25 mmol, 0.07 g), and dipotassium hydrogenphosphate (0.22 mmol, 0.05 g) in 10 ml distilled water was sealed in a 25 ml Teflon-lined stainless steel autoclave and was kept at 433 K for three days. Brown crystals suitable for the X-ray experiment were obtained. IR(cm^-1^): 3441, 3118, 2674, 2546, 1696, 1605, 1540, 1429, 1356, 1291, 1107, 1014, 931, 876, 802, 719, 562, 507.

TGA curve shows a separation of lattice water molecules and the organic ligands above 352 and 655 K, respectively. The overall thermal decomposition process can be described by the followed equation: 4C_48_H_54_Fe_2_Mo_12_N_18_O_46_P + 327O_2_ = 108H_2_O + 192CO_2_ + 36N_2_O_5_ + 4Fe_2_O_3_ + 2P_2_O_5_ + 48MoO_3_

### Refinement

All hydrogen atoms bound to aromatic carbon atoms were refined in calculated positions using a riding model with a C—H distance of 0.93 Å and *U*_iso_ = 1.2*U*_eq_(C). Hydrogen atoms attached to aromatic N atoms were refined with a N—H distance of 0.86 Å and *U*_iso_ = 1.2*U*_eq_(N). The hydrogen atoms of the three uncoordinated water molecules could not be located unambiguously from difference Fourier maps, probably due to disorder of the water molecules. Thus the structure was refined without the H atoms of the water molecules (which includes the water O atoms O1W, O2W, O3W). In the PO_4_ unit, the two oxygen atoms (O19 and O21) are equally disordered about the inversion centre. In the final difference Fourier map the highest peak is 2.82 Å from atom O2w and the deepest hole is 0.79 Å from atom Mo1. The highest peak is located in the voids of the crystal structure and may be associated with an additional water molecule. However, refinement of this position did not result in a reasonable model. Hence this position was also excluded from the final refinement.

### Crystal data

**Table:**

[(Fe(C_8_H_7_N_3_)_3_]_2_[PMo_12_O_40_]·6H_2_O	*F*(000) = 5628
*M**_r_* = 2913.04	*D*_x_ = 2.384 Mg m^−^^3^
Monoclinic, *C*2/*c*	Mo *K*α radiation, λ = 0.71073 Å
Hall symbol: -C 2yc	Cell parameters from 9828 reflections
*a* = 18.6816 (10) Å	θ = 2.4–28.2°
*b* = 16.2224 (10) Å	µ = 2.26 mm^−^^1^
*c* = 27.6405 (15) Å	*T* = 293 K
β = 104.295 (1)°	Block, brown
*V* = 8117.4 (8) Å^3^	0.12 × 0.10 × 0.08 mm
*Z* = 4	

### Data collection

**Table:**

Bruker APEXII CCD diffractometer	7090 independent reflections
Radiation source: fine-focus sealed tube	5550 reflections with *I* > 2σ(*I*)
graphite	*R*_int_ = 0.042
phi and ω scans	θ_max_ = 25.0°, θ_min_ = 1.5°
Absorption correction: multi-scan (*SADABS*; Bruker, 2001)	*h* = −22→17
*T*_min_ = 0.773, *T*_max_ = 0.840	*k* = −18→19
20565 measured reflections	*l* = −32→31

### Refinement

**Table:**

Refinement on *F*^2^	Primary atom site location: structure-invariant direct methods
Least-squares matrix: full	Secondary atom site location: difference Fourier map
*R*[*F*^2^ > 2σ(*F*^2^)] = 0.039	Hydrogen site location: inferred from neighbouring sites
*wR*(*F*^2^) = 0.142	H-atom parameters constrained
*S* = 1.00	*w* = 1/[σ^2^(*F*_o_^2^) + (0.1*P*)^2^ + 20.1545*P*] where *P* = (*F*_o_^2^ + 2*F*_c_^2^)/3
7090 reflections	(Δ/σ)_max_ = 0.001
592 parameters	Δρ_max_ = 1.36 e Å^−^^3^
0 restraints	Δρ_min_ = −0.80 e Å^−^^3^

### Special details

**Table:**

Geometry. All esds (except the esd in the dihedral angle between two l.s. planes) are estimated using the full covariance matrix. The cell esds are taken into account individually in the estimation of esds in distances, angles and torsion angles; correlations between esds in cell parameters are only used when they are defined by crystal symmetry. An approximate (isotropic) treatment of cell esds is used for estimating esds involving l.s. planes.
Refinement. Refinement of *F*^2^ against ALL reflections. The weighted *R*-factor wR and goodness of fit *S* are based on *F*^2^, conventional *R*-factors *R* are based on *F*, with *F* set to zero for negative *F*^2^. The threshold expression of *F*^2^ > σ(*F*^2^) is used only for calculating *R*-factors(gt) etc. and is not relevant to the choice of reflections for refinement. *R*-factors based on *F*^2^ are statistically about twice as large as those based on *F*, and *R*- factors based on ALL data will be even larger.

### Fractional atomic coordinates and isotropic or equivalent isotropic displacement parameters (Å^2^)

**Table:**

	*x*	*y*	*z*	*U*_iso_*/*U*_eq_	Occ. (<1)
C1	0.2037 (13)	0.8989 (13)	0.0951 (8)	0.049 (5)	
H1	0.2470	0.8948	0.0844	0.059*	
C2	0.1427 (14)	0.9335 (14)	0.0638 (8)	0.054 (6)	
H2	0.1446	0.9517	0.0323	0.065*	
C3	0.0793 (13)	0.9410 (15)	0.0791 (8)	0.055 (6)	
H3	0.0379	0.9660	0.0588	0.065*	
C4	0.0780 (13)	0.9107 (15)	0.1252 (9)	0.055 (6)	
H4	0.0345	0.9123	0.1359	0.066*	
C5	0.1406 (12)	0.8781 (12)	0.1553 (7)	0.042 (5)	
C6	0.1448 (12)	0.8483 (13)	0.2053 (8)	0.045 (5)	
C7	0.0917 (16)	0.845 (2)	0.2338 (10)	0.076 (8)	
H7	0.0423	0.8603	0.2244	0.091*	
C8	0.1307 (19)	0.814 (2)	0.2788 (9)	0.079 (9)	
H8	0.1119	0.8047	0.3065	0.095*	
C9	0.3237 (16)	0.9793 (18)	0.2596 (11)	0.071 (7)	
H9	0.2903	0.9589	0.2766	0.085*	
C10	0.3526 (18)	1.0535 (19)	0.2716 (12)	0.076 (8)	
H10	0.3402	1.0833	0.2971	0.091*	
C11	0.400 (2)	1.086 (3)	0.2472 (15)	0.098 (11)	
H11	0.4198	1.1378	0.2557	0.118*	
C12	0.4175 (17)	1.043 (2)	0.2108 (14)	0.090 (10)	
H12	0.4493	1.0655	0.1931	0.108*	
C13	0.3882 (13)	0.9665 (18)	0.2001 (11)	0.066 (7)	
C14	0.4075 (15)	0.915 (2)	0.1621 (11)	0.074 (8)	
C15	0.4577 (19)	0.926 (3)	0.1312 (14)	0.101 (11)	
H15	0.4894	0.9699	0.1306	0.121*	
C16	0.4475 (19)	0.853 (2)	0.1010 (13)	0.091 (10)	
H16	0.4719	0.8392	0.0766	0.109*	
C17	0.4084 (15)	0.758 (2)	0.2901 (12)	0.077 (8)	
H17	0.4135	0.8138	0.2988	0.093*	
C18	0.4475 (19)	0.701 (2)	0.3232 (13)	0.097 (11)	
H18	0.4766	0.7174	0.3540	0.116*	
C19	0.443 (2)	0.622 (2)	0.3106 (14)	0.103 (12)	
H19	0.4709	0.5832	0.3319	0.123*	
C20	0.3977 (17)	0.5978 (18)	0.2657 (11)	0.078 (8)	
H20	0.3943	0.5426	0.2563	0.093*	
C21	0.3579 (13)	0.6562 (15)	0.2356 (9)	0.054 (6)	
C22	0.3055 (13)	0.6380 (14)	0.1877 (9)	0.054 (6)	
C23	0.2857 (17)	0.5644 (17)	0.1626 (10)	0.069 (7)	
H23	0.3049	0.5122	0.1716	0.083*	
C24	0.2312 (19)	0.5861 (18)	0.1213 (11)	0.077 (8)	
H24	0.2051	0.5507	0.0968	0.092*	
Fe1	0.29393 (17)	0.8180 (2)	0.19341 (13)	0.0479 (8)	
Mo1	0.24259 (11)	0.14187 (11)	0.11115 (6)	0.0414 (5)	
Mo2	0.19058 (10)	0.45820 (10)	−0.01680 (7)	0.0420 (5)	
Mo3	0.42269 (9)	0.31452 (11)	−0.01975 (7)	0.0386 (5)	
Mo4	0.35343 (9)	0.40933 (11)	0.07504 (6)	0.0398 (5)	
Mo5	0.41686 (9)	0.20224 (11)	0.09088 (6)	0.0405 (5)	
Mo6	0.17783 (10)	0.34811 (12)	0.09231 (6)	0.0420 (5)	
N1	0.2034 (9)	0.8711 (11)	0.1403 (6)	0.042 (4)	
N2	0.2095 (10)	0.8208 (12)	0.2318 (7)	0.051 (5)	
N3	0.1991 (13)	0.7990 (14)	0.2763 (7)	0.068 (6)	
H3A	0.2327	0.7779	0.3001	0.081*	
N4	0.3407 (12)	0.9323 (13)	0.2238 (8)	0.064 (6)	
N5	0.3703 (13)	0.8449 (17)	0.1521 (8)	0.072 (7)	
N6	0.3958 (16)	0.8079 (19)	0.1153 (10)	0.093 (9)	
H6	0.3805	0.7608	0.1026	0.112*	
N7	0.3639 (11)	0.7377 (13)	0.2466 (8)	0.057 (5)	
N8	0.2677 (11)	0.7008 (11)	0.1633 (7)	0.052 (5)	
N9	0.2227 (12)	0.6693 (13)	0.1233 (8)	0.061 (5)	
H9A	0.1918	0.6975	0.1013	0.073*	
O1	0.4070 (10)	0.3947 (9)	0.0244 (6)	0.062 (5)	
O2	0.1470 (9)	0.3952 (11)	0.1364 (6)	0.059 (4)	
O3	0.2402 (9)	0.0913 (10)	0.1620 (5)	0.056 (4)	
O4	0.3455 (11)	0.1738 (12)	0.1246 (7)	0.077 (6)	
O5	0.4049 (11)	0.3141 (10)	0.1060 (6)	0.065 (5)	
O6	0.2789 (8)	0.3892 (13)	0.1084 (7)	0.071 (5)	
O7	0.4527 (11)	0.2401 (10)	0.0354 (6)	0.065 (5)	
O8	0.4029 (10)	0.4828 (10)	0.1090 (6)	0.062 (4)	
O9	0.4022 (12)	0.2143 (10)	−0.0602 (7)	0.085 (7)	
O10	0.5038 (9)	0.3410 (10)	−0.0293 (7)	0.062 (4)	
O11	0.2853 (9)	0.4702 (15)	0.0264 (7)	0.083 (7)	
O12	0.1036 (11)	0.4003 (12)	−0.0543 (7)	0.083 (7)	
O13	0.1436 (12)	0.1335 (10)	0.0730 (8)	0.088 (7)	
O14	0.1569 (9)	0.4260 (15)	0.0409 (7)	0.084 (6)	
O15	0.1641 (9)	0.5548 (9)	−0.0236 (7)	0.064 (5)	
O16	0.2690 (11)	0.0585 (13)	0.0706 (8)	0.087 (7)	
O17	0.2216 (10)	0.2520 (10)	0.1270 (7)	0.078 (6)	
O18	0.4938 (9)	0.1788 (12)	0.1315 (6)	0.066 (5)	
O19A	0.2032 (13)	0.2368 (14)	0.0391 (8)	0.030 (5)	0.50
O21A	0.3250 (13)	0.2802 (15)	0.0252 (9)	0.030 (5)	0.50
O19B	0.2909 (13)	0.1861 (14)	0.0380 (8)	0.028 (5)	0.50
O21B	0.2480 (14)	0.3317 (15)	0.0266 (9)	0.032 (5)	0.50
O1W	0.1172 (13)	0.7180 (15)	0.0392 (9)	0.096 (7)	
O2W	0.362 (3)	0.657 (2)	0.0638 (17)	0.186 (11)	
O3W	0.534 (2)	0.071 (3)	0.0275 (19)	0.199 (13)	
P1	0.2500	0.2500	0.0000	0.0217 (12)	

### Atomic displacement parameters (Å^2^)

**Table:**

	*U*^11^	*U*^22^	*U*^33^	*U*^12^	*U*^13^	*U*^23^
C1	0.047 (12)	0.048 (12)	0.051 (13)	−0.004 (10)	0.012 (10)	0.016 (10)
C2	0.064 (15)	0.048 (12)	0.046 (12)	−0.015 (12)	0.007 (11)	0.009 (10)
C3	0.041 (12)	0.062 (14)	0.047 (13)	−0.004 (11)	−0.014 (10)	0.011 (11)
C4	0.039 (12)	0.070 (15)	0.055 (14)	0.001 (11)	0.008 (10)	0.004 (12)
C5	0.043 (11)	0.040 (11)	0.040 (11)	−0.002 (9)	0.005 (9)	0.002 (9)
C6	0.037 (11)	0.053 (12)	0.044 (12)	−0.006 (10)	0.006 (9)	0.003 (10)
C7	0.063 (17)	0.11 (2)	0.067 (17)	0.011 (16)	0.035 (14)	0.013 (16)
C8	0.09 (2)	0.11 (2)	0.037 (13)	−0.009 (19)	0.020 (14)	0.018 (14)
C9	0.070 (18)	0.070 (17)	0.080 (19)	0.018 (15)	0.033 (15)	0.027 (15)
C10	0.08 (2)	0.071 (19)	0.076 (19)	0.009 (16)	0.014 (15)	0.006 (15)
C11	0.08 (2)	0.10 (3)	0.10 (3)	0.01 (2)	0.00 (2)	0.02 (2)
C12	0.051 (17)	0.11 (3)	0.10 (2)	−0.010 (18)	−0.005 (17)	0.02 (2)
C13	0.036 (12)	0.077 (18)	0.076 (17)	0.007 (12)	−0.001 (12)	0.032 (14)
C14	0.049 (15)	0.10 (2)	0.070 (18)	0.022 (16)	0.016 (13)	0.038 (17)
C15	0.068 (17)	0.13 (2)	0.11 (2)	0.018 (17)	0.018 (16)	0.051 (19)
C16	0.082 (18)	0.12 (2)	0.082 (18)	0.038 (17)	0.050 (15)	0.028 (17)
C17	0.053 (16)	0.08 (2)	0.09 (2)	−0.001 (15)	0.006 (15)	−0.021 (17)
C18	0.07 (2)	0.10 (3)	0.09 (2)	−0.006 (19)	−0.027 (18)	0.00 (2)
C19	0.09 (3)	0.09 (2)	0.10 (3)	0.02 (2)	−0.02 (2)	0.01 (2)
C20	0.074 (19)	0.065 (17)	0.08 (2)	0.012 (14)	−0.002 (16)	0.008 (15)
C21	0.044 (12)	0.054 (14)	0.064 (15)	0.003 (11)	0.016 (11)	0.000 (11)
C22	0.054 (14)	0.049 (13)	0.061 (14)	0.010 (11)	0.021 (11)	−0.004 (11)
C23	0.09 (2)	0.053 (14)	0.066 (17)	0.008 (14)	0.018 (15)	−0.002 (13)
C24	0.10 (2)	0.067 (18)	0.063 (17)	0.006 (16)	0.015 (16)	−0.017 (13)
Fe1	0.0393 (17)	0.0462 (18)	0.060 (2)	0.0076 (14)	0.0151 (14)	0.0097 (14)
Mo1	0.0516 (11)	0.0396 (10)	0.0317 (9)	−0.0035 (8)	0.0077 (8)	0.0034 (7)
Mo2	0.0421 (10)	0.0313 (9)	0.0549 (11)	0.0073 (7)	0.0165 (8)	0.0039 (7)
Mo3	0.0257 (9)	0.0415 (10)	0.0474 (11)	−0.0038 (7)	0.0068 (7)	−0.0020 (8)
Mo4	0.0308 (9)	0.0425 (10)	0.0436 (10)	−0.0059 (7)	0.0044 (7)	−0.0096 (7)
Mo5	0.0281 (9)	0.0501 (11)	0.0394 (10)	0.0028 (7)	0.0006 (7)	0.0023 (8)
Mo6	0.0423 (10)	0.0483 (11)	0.0350 (10)	0.0105 (8)	0.0087 (8)	−0.0084 (7)
N1	0.036 (9)	0.047 (10)	0.043 (10)	−0.002 (8)	0.010 (7)	0.005 (8)
N2	0.043 (10)	0.056 (11)	0.044 (10)	−0.007 (9)	−0.005 (8)	0.010 (8)
N3	0.073 (16)	0.082 (15)	0.040 (11)	−0.016 (12)	−0.001 (10)	0.020 (10)
N4	0.058 (13)	0.063 (13)	0.078 (14)	0.019 (11)	0.029 (11)	0.027 (11)
N5	0.062 (14)	0.098 (18)	0.063 (14)	0.034 (14)	0.027 (11)	0.022 (13)
N6	0.09 (2)	0.12 (2)	0.074 (16)	0.054 (18)	0.024 (15)	0.018 (15)
N7	0.040 (10)	0.067 (13)	0.064 (13)	−0.002 (9)	0.010 (9)	−0.007 (10)
N8	0.051 (11)	0.051 (11)	0.050 (11)	0.007 (9)	0.006 (9)	−0.004 (9)
N9	0.071 (14)	0.056 (12)	0.051 (12)	0.003 (10)	0.007 (10)	−0.006 (9)
O1	0.094 (13)	0.045 (9)	0.059 (10)	0.024 (9)	0.042 (9)	0.009 (7)
O2	0.048 (9)	0.081 (12)	0.048 (9)	0.008 (8)	0.013 (7)	−0.022 (8)
O3	0.057 (10)	0.069 (10)	0.042 (8)	−0.010 (8)	0.009 (7)	0.012 (7)
O4	0.071 (12)	0.086 (13)	0.089 (13)	−0.046 (10)	0.049 (11)	−0.049 (11)
O5	0.101 (14)	0.048 (9)	0.062 (10)	0.026 (9)	0.049 (10)	0.012 (8)
O6	0.030 (8)	0.114 (15)	0.067 (11)	0.004 (9)	0.010 (7)	0.040 (10)
O7	0.099 (14)	0.051 (9)	0.055 (10)	0.025 (9)	0.040 (9)	0.011 (8)
O8	0.075 (12)	0.049 (9)	0.058 (10)	−0.020 (9)	0.009 (8)	−0.014 (8)
O9	0.092 (13)	0.039 (8)	0.087 (12)	0.007 (9)	−0.046 (10)	−0.015 (8)
O10	0.047 (9)	0.065 (10)	0.085 (12)	−0.003 (8)	0.038 (9)	−0.001 (9)
O11	0.038 (9)	0.146 (19)	0.068 (11)	0.010 (11)	0.015 (8)	0.052 (12)
O12	0.078 (13)	0.092 (14)	0.096 (14)	−0.049 (11)	0.055 (11)	−0.052 (11)
O13	0.086 (14)	0.043 (9)	0.096 (14)	0.018 (9)	−0.053 (11)	−0.021 (9)
O14	0.037 (9)	0.137 (18)	0.074 (12)	−0.004 (10)	0.007 (8)	0.054 (12)
O15	0.052 (10)	0.036 (8)	0.101 (13)	0.014 (7)	0.012 (9)	0.006 (8)
O16	0.079 (13)	0.097 (14)	0.105 (14)	−0.053 (12)	0.060 (12)	−0.062 (12)
O17	0.069 (12)	0.046 (9)	0.088 (13)	0.007 (8)	−0.038 (10)	−0.013 (9)
O18	0.051 (10)	0.094 (13)	0.043 (9)	0.029 (9)	−0.007 (7)	0.004 (8)
O19A	0.023 (12)	0.037 (13)	0.028 (12)	0.002 (10)	0.004 (10)	−0.005 (10)
O21A	0.022 (12)	0.033 (13)	0.036 (13)	−0.001 (10)	0.008 (10)	−0.001 (10)
O19B	0.032 (13)	0.025 (11)	0.025 (12)	0.001 (10)	0.003 (10)	−0.003 (9)
O21B	0.035 (14)	0.032 (13)	0.029 (13)	0.000 (11)	0.006 (10)	−0.001 (10)
O1W	0.077 (14)	0.102 (16)	0.095 (16)	0.019 (13)	−0.005 (12)	0.004 (13)
O2W	0.231 (11)	0.150 (11)	0.239 (11)	−0.014 (3)	0.179 (4)	−0.011 (3)
O3W	0.166 (13)	0.165 (13)	0.311 (13)	0.000 (3)	0.140 (5)	−0.042 (3)
P1	0.022 (3)	0.020 (3)	0.022 (3)	0.002 (2)	0.004 (2)	−0.001 (2)

### Geometric parameters (Å, °)

**Table:**

C1—N1	1.33 (3)	Mo1—O19B	2.51 (2)
C1—C2	1.37 (3)	Mo2—O15	1.641 (14)
C1—H1	0.9300	Mo2—O16^i^	1.847 (17)
C2—C3	1.36 (3)	Mo2—O11	1.884 (18)
C2—H2	0.9300	Mo2—O14	1.925 (17)
C3—C4	1.37 (3)	Mo2—O12	1.941 (18)
C3—H3	0.9300	Mo2—O19B^i^	2.46 (2)
C4—C5	1.36 (3)	Mo2—O21B	2.48 (2)
C4—H4	0.9300	Mo3—O10	1.659 (15)
C5—N1	1.34 (3)	Mo3—O1	1.855 (15)
C5—C6	1.45 (3)	Mo3—O13^i^	1.875 (16)
C6—N2	1.33 (3)	Mo3—O7	1.916 (15)
C6—C7	1.41 (3)	Mo3—O9	1.958 (16)
C7—C8	1.37 (4)	Mo3—O19A^i^	2.43 (2)
C7—H7	0.9300	Mo3—O21A	2.51 (2)
C8—N3	1.32 (4)	Mo4—O8	1.652 (15)
C8—H8	0.9300	Mo4—O6	1.878 (16)
C9—C10	1.33 (4)	Mo4—O11	1.887 (17)
C9—N4	1.35 (3)	Mo4—O5	1.907 (16)
C9—H9	0.9300	Mo4—O1	1.926 (15)
C10—C11	1.34 (5)	Mo4—O21B	2.44 (2)
C10—H10	0.9300	Mo4—O21A	2.49 (2)
C11—C12	1.33 (5)	Mo5—O18	1.636 (15)
C11—H11	0.9300	Mo5—O4	1.864 (16)
C12—C13	1.36 (4)	Mo5—O5	1.888 (15)
C12—H12	0.9300	Mo5—O7	1.920 (15)
C13—N4	1.35 (3)	Mo5—O12^i^	1.935 (17)
C13—C14	1.45 (4)	Mo5—O19B	2.46 (2)
C14—N5	1.33 (4)	Mo5—O21A	2.51 (2)
C14—C15	1.43 (4)	Mo6—O2	1.657 (14)
C15—C16	1.43 (5)	Mo6—O9^i^	1.841 (17)
C15—H15	0.9300	Mo6—O14	1.870 (17)
C16—N6	1.34 (4)	Mo6—O17	1.907 (17)
C16—H16	0.9300	Mo6—O6	1.948 (16)
C17—N7	1.33 (3)	Mo6—O19A	2.45 (2)
C17—C18	1.38 (4)	Mo6—O21B	2.50 (2)
C17—H17	0.9300	N2—N3	1.34 (3)
C18—C19	1.32 (5)	N3—H3A	0.8600
C18—H18	0.9300	N5—N6	1.37 (3)
C19—C20	1.38 (4)	N6—H6	0.8600
C19—H19	0.9300	N8—N9	1.32 (3)
C20—C21	1.36 (3)	N9—H9A	0.8600
C20—H20	0.9300	O9—Mo6^i^	1.841 (17)
C21—N7	1.36 (3)	O12—Mo5^i^	1.935 (17)
C21—C22	1.47 (4)	O13—Mo3^i^	1.875 (16)
C22—N8	1.33 (3)	O16—Mo2^i^	1.847 (17)
C22—C23	1.38 (3)	O19A—P1	1.56 (2)
C23—C24	1.37 (4)	O19A—O21A^i^	1.75 (3)
C23—H23	0.9300	O19A—Mo3^i^	2.43 (2)
C24—N9	1.36 (3)	O21A—P1	1.49 (2)
C24—H24	0.9300	O21A—O21B	1.67 (3)
Fe1—N8	2.085 (19)	O21A—O19B	1.72 (3)
Fe1—N5	2.08 (2)	O21A—O19A^i^	1.75 (3)
Fe1—N2	2.11 (2)	O19B—P1	1.54 (2)
Fe1—N1	2.129 (17)	O19B—O21B^i^	1.78 (3)
Fe1—N4	2.13 (2)	O19B—Mo2^i^	2.46 (2)
Fe1—N7	2.15 (2)	O21B—P1	1.52 (2)
Mo1—O3	1.639 (14)	O21B—O19B^i^	1.78 (3)
Mo1—O13	1.893 (18)	P1—O21A^i^	1.49 (2)
Mo1—O16	1.898 (17)	P1—O21B^i^	1.52 (2)
Mo1—O17	1.903 (17)	P1—O19B^i^	1.54 (2)
Mo1—O4	1.937 (17)	P1—O19A^i^	1.56 (2)
Mo1—O19A	2.48 (2)		
			
N1—C1—C2	122 (2)	O18—Mo5—O4	102.4 (10)
N1—C1—H1	118.8	O18—Mo5—O5	101.7 (9)
C2—C1—H1	118.8	O4—Mo5—O5	89.4 (8)
C1—C2—C3	120 (2)	O18—Mo5—O7	101.6 (9)
C1—C2—H2	120.2	O4—Mo5—O7	155.9 (9)
C3—C2—H2	120.2	O5—Mo5—O7	87.3 (7)
C2—C3—C4	118 (2)	O18—Mo5—O12^i^	100.7 (10)
C2—C3—H3	120.8	O4—Mo5—O12^i^	88.6 (7)
C4—C3—H3	120.9	O5—Mo5—O12^i^	157.4 (9)
C5—C4—C3	120 (2)	O7—Mo5—O12^i^	85.4 (7)
C5—C4—H4	120.2	O18—Mo5—O19B	159.0 (9)
C3—C4—H4	120.2	O4—Mo5—O19B	64.8 (8)
N1—C5—C4	122 (2)	O5—Mo5—O19B	94.9 (8)
N1—C5—C6	114.9 (18)	O7—Mo5—O19B	91.7 (8)
C4—C5—C6	123 (2)	O12^i^—Mo5—O19B	64.0 (8)
N2—C6—C7	110 (2)	O18—Mo5—O21A	160.3 (9)
N2—C6—C5	117.8 (19)	O4—Mo5—O21A	92.4 (9)
C7—C6—C5	132 (2)	O5—Mo5—O21A	65.1 (8)
C8—C7—C6	103 (3)	O7—Mo5—O21A	64.6 (8)
C8—C7—H7	128.3	O12^i^—Mo5—O21A	92.5 (9)
C6—C7—H7	128.3	O19B—Mo5—O21A	40.6 (7)
N3—C8—C7	109 (2)	O2—Mo6—O9^i^	102.7 (9)
N3—C8—H8	125.6	O2—Mo6—O14	101.7 (9)
C7—C8—H8	125.6	O9^i^—Mo6—O14	90.7 (9)
C10—C9—N4	123 (3)	O2—Mo6—O17	101.0 (9)
C10—C9—H9	118.7	O9^i^—Mo6—O17	89.4 (7)
N4—C9—H9	118.6	O14—Mo6—O17	156.7 (10)
C9—C10—C11	120 (3)	O2—Mo6—O6	99.7 (8)
C9—C10—H10	119.8	O9^i^—Mo6—O6	157.5 (10)
C11—C10—H10	119.8	O14—Mo6—O6	87.2 (7)
C12—C11—C10	120 (4)	O17—Mo6—O6	83.9 (8)
C12—C11—H11	120.0	O2—Mo6—O19A	159.3 (8)
C10—C11—H11	120.1	O9^i^—Mo6—O19A	63.9 (9)
C11—C12—C13	118 (4)	O14—Mo6—O19A	94.4 (9)
C11—C12—H12	120.8	O17—Mo6—O19A	64.9 (8)
C13—C12—H12	120.8	O6—Mo6—O19A	94.0 (8)
N4—C13—C12	123 (3)	O2—Mo6—O21B	157.2 (8)
N4—C13—C14	115 (3)	O9^i^—Mo6—O21B	95.8 (9)
C12—C13—C14	121 (3)	O14—Mo6—O21B	64.5 (9)
N5—C14—C15	111 (3)	O17—Mo6—O21B	92.4 (9)
N5—C14—C13	116 (2)	O6—Mo6—O21B	63.2 (8)
C15—C14—C13	133 (3)	O19A—Mo6—O21B	43.3 (8)
C14—C15—C16	104 (3)	C1—N1—C5	117.9 (18)
C14—C15—H15	128.1	C1—N1—Fe1	126.7 (15)
C16—C15—H15	128.2	C5—N1—Fe1	115.4 (13)
N6—C16—C15	106 (3)	C6—N2—N3	106 (2)
N6—C16—H16	126.9	C6—N2—Fe1	115.0 (14)
C15—C16—H16	126.8	N3—N2—Fe1	138.8 (16)
N7—C17—C18	123 (3)	C8—N3—N2	111 (2)
N7—C17—H17	118.6	C8—N3—H3A	124.4
C18—C17—H17	118.6	N2—N3—H3A	124.4
C19—C18—C17	119 (3)	C9—N4—C13	115 (3)
C19—C18—H18	120.3	C9—N4—Fe1	129.3 (19)
C17—C18—H18	120.3	C13—N4—Fe1	115 (2)
C18—C19—C20	120 (3)	C14—N5—N6	106 (3)
C18—C19—H19	120.0	C14—N5—Fe1	117.3 (19)
C20—C19—H19	120.1	N6—N5—Fe1	137 (2)
C21—C20—C19	118 (3)	N5—N6—C16	113 (3)
C21—C20—H20	120.8	N5—N6—H6	123.6
C19—C20—H20	120.8	C16—N6—H6	123.5
N7—C21—C20	123 (2)	C17—N7—C21	117 (2)
N7—C21—C22	113 (2)	C17—N7—Fe1	127.5 (19)
C20—C21—C22	124 (2)	C21—N7—Fe1	115.8 (16)
N8—C22—C23	111 (2)	N9—N8—C22	106.2 (19)
N8—C22—C21	117 (2)	N9—N8—Fe1	136.8 (15)
C23—C22—C21	131 (2)	C22—N8—Fe1	117.0 (16)
C24—C23—C22	104 (2)	N8—N9—C24	111 (2)
C24—C23—H23	127.8	N8—N9—H9A	124.4
C22—C23—H23	127.8	C24—N9—H9A	124.4
N9—C24—C23	107 (3)	Mo3—O1—Mo4	139.4 (10)
N9—C24—H24	126.6	Mo5—O4—Mo1	140.2 (11)
C23—C24—H24	126.5	Mo5—O5—Mo4	138.8 (11)
N8—Fe1—N5	95.6 (10)	Mo4—O6—Mo6	137.9 (11)
N8—Fe1—N2	95.1 (8)	Mo3—O7—Mo5	138.2 (10)
N5—Fe1—N2	166.1 (9)	Mo6^i^—O9—Mo3	138.8 (13)
N8—Fe1—N1	91.4 (7)	Mo2—O11—Mo4	139.5 (13)
N5—Fe1—N1	94.2 (7)	Mo5^i^—O12—Mo2	136.6 (12)
N2—Fe1—N1	76.8 (6)	Mo3^i^—O13—Mo1	140.3 (13)
N8—Fe1—N4	169.6 (8)	Mo6—O14—Mo2	139.8 (12)
N5—Fe1—N4	76.3 (10)	Mo2^i^—O16—Mo1	142.9 (12)
N2—Fe1—N4	93.9 (7)	Mo1—O17—Mo6	137.6 (11)
N1—Fe1—N4	95.7 (7)	P1—O19A—O21A^i^	53.0 (10)
N8—Fe1—N7	76.4 (8)	P1—O19A—Mo3^i^	124.8 (12)
N5—Fe1—N7	96.8 (8)	O21A^i^—O19A—Mo3^i^	71.9 (11)
N2—Fe1—N7	94.2 (7)	P1—O19A—Mo6	122.6 (13)
N1—Fe1—N7	164.3 (7)	O21A^i^—O19A—Mo6	132.3 (14)
N4—Fe1—N7	97.8 (8)	Mo3^i^—O19A—Mo6	93.7 (8)
O3—Mo1—O13	102.2 (9)	P1—O19A—Mo1	122.3 (12)
O3—Mo1—O16	102.5 (9)	O21A^i^—O19A—Mo1	132.4 (14)
O13—Mo1—O16	88.9 (9)	Mo3^i^—O19A—Mo1	92.5 (8)
O3—Mo1—O17	102.8 (9)	Mo6—O19A—Mo1	92.2 (8)
O13—Mo1—O17	88.2 (7)	P1—O21A—O21B	57.2 (12)
O16—Mo1—O17	154.6 (10)	P1—O21A—O19B	56.7 (11)
O3—Mo1—O4	101.8 (8)	O21B—O21A—O19B	94.2 (16)
O13—Mo1—O4	155.9 (10)	P1—O21A—O19A^i^	57.2 (11)
O16—Mo1—O4	85.1 (7)	O21B—O21A—O19A^i^	93.2 (16)
O17—Mo1—O4	87.5 (8)	O19B—O21A—O19A^i^	92.0 (16)
O3—Mo1—O19A	159.1 (8)	P1—O21A—Mo4	125.7 (13)
O13—Mo1—O19A	62.8 (9)	O21B—O21A—Mo4	68.6 (11)
O16—Mo1—O19A	92.1 (9)	O19B—O21A—Mo4	132.1 (14)
O17—Mo1—O19A	64.2 (8)	O19A^i^—O21A—Mo4	131.7 (15)
O4—Mo1—O19A	94.2 (8)	P1—O21A—Mo5	124.8 (13)
O3—Mo1—O19B	157.6 (8)	O21B—O21A—Mo5	132.4 (15)
O13—Mo1—O19B	93.9 (9)	O19B—O21A—Mo5	68.1 (11)
O16—Mo1—O19B	62.0 (8)	O19A^i^—O21A—Mo5	129.6 (14)
O17—Mo1—O19B	93.0 (9)	Mo4—O21A—Mo5	90.6 (8)
O4—Mo1—O19B	62.7 (8)	P1—O21A—Mo3	123.9 (13)
O19A—Mo1—O19B	43.3 (7)	O21B—O21A—Mo3	129.4 (15)
O15—Mo2—O16^i^	102.7 (10)	O19B—O21A—Mo3	130.4 (14)
O15—Mo2—O11	100.6 (9)	O19A^i^—O21A—Mo3	66.7 (10)
O16^i^—Mo2—O11	91.0 (9)	Mo4—O21A—Mo3	90.3 (8)
O15—Mo2—O14	101.7 (10)	Mo5—O21A—Mo3	91.1 (8)
O16^i^—Mo2—O14	155.4 (10)	P1—O19B—O21A	53.8 (11)
O11—Mo2—O14	87.2 (7)	P1—O19B—O21B^i^	54.0 (10)
O15—Mo2—O12	102.3 (9)	O21A—O19B—O21B^i^	91.1 (15)
O16^i^—Mo2—O12	87.4 (7)	P1—O19B—Mo2^i^	123.7 (12)
O11—Mo2—O12	156.9 (10)	O21A—O19B—Mo2^i^	135.5 (14)
O14—Mo2—O12	84.8 (8)	O21B^i^—O19B—Mo2^i^	69.7 (10)
O15—Mo2—O19B^i^	159.9 (8)	P1—O19B—Mo5	125.1 (12)
O16^i^—Mo2—O19B^i^	63.8 (8)	O21A—O19B—Mo5	71.3 (11)
O11—Mo2—O19B^i^	94.8 (9)	O21B^i^—O19B—Mo5	135.2 (15)
O14—Mo2—O19B^i^	91.9 (9)	Mo2^i^—O19B—Mo5	94.2 (8)
O12—Mo2—O19B^i^	63.9 (8)	P1—O19B—Mo1	121.7 (12)
O15—Mo2—O21B	158.0 (9)	O21A—O19B—Mo1	129.9 (13)
O16^i^—Mo2—O21B	93.0 (10)	O21B^i^—O19B—Mo1	128.5 (14)
O11—Mo2—O21B	63.5 (9)	Mo2^i^—O19B—Mo1	91.1 (7)
O14—Mo2—O21B	64.3 (9)	Mo5—O19B—Mo1	91.9 (7)
O12—Mo2—O21B	93.6 (9)	P1—O21B—O21A	55.3 (11)
O19B^i^—Mo2—O21B	42.1 (8)	P1—O21B—O19B^i^	55.0 (11)
O10—Mo3—O1	102.8 (8)	O21A—O21B—O19B^i^	92.2 (16)
O10—Mo3—O13^i^	102.2 (10)	P1—O21B—Mo4	127.1 (14)
O1—Mo3—O13^i^	91.1 (8)	O21A—O21B—Mo4	71.8 (12)
O10—Mo3—O7	101.0 (9)	O19B^i^—O21B—Mo4	135.2 (15)
O1—Mo3—O7	89.0 (7)	P1—O21B—Mo2	123.1 (13)
O13^i^—Mo3—O7	156.2 (10)	O21A—O21B—Mo2	132.4 (15)
O10—Mo3—O9	100.5 (10)	O19B^i^—O21B—Mo2	68.2 (10)
O1—Mo3—O9	156.6 (9)	Mo4—O21B—Mo2	91.9 (8)
O13^i^—Mo3—O9	85.8 (7)	P1—O21B—Mo6	121.5 (13)
O7—Mo3—O9	84.8 (8)	O21A—O21B—Mo6	132.6 (15)
O10—Mo3—O19A^i^	158.1 (8)	O19B^i^—O21B—Mo6	126.0 (15)
O1—Mo3—O19A^i^	94.8 (8)	Mo4—O21B—Mo6	92.4 (8)
O13^i^—Mo3—O19A^i^	64.2 (9)	Mo2—O21B—Mo6	91.2 (8)
O7—Mo3—O19A^i^	92.1 (8)	O21A^i^—P1—O21A	180 (3)
O9—Mo3—O19A^i^	63.0 (9)	O21A^i^—P1—O21B^i^	67.6 (13)
O10—Mo3—O21A	160.2 (8)	O21A—P1—O21B^i^	112.4 (13)
O1—Mo3—O21A	65.0 (8)	O21A^i^—P1—O21B	112.4 (13)
O13^i^—Mo3—O21A	93.9 (10)	O21A—P1—O21B	67.6 (13)
O7—Mo3—O21A	64.7 (8)	O21B^i^—P1—O21B	180 (2)
O9—Mo3—O21A	92.0 (9)	O21A^i^—P1—O19B	110.5 (12)
O19A^i^—Mo3—O21A	41.4 (8)	O21A—P1—O19B	69.5 (12)
O8—Mo4—O6	103.0 (9)	O21B^i^—P1—O19B	71.0 (12)
O8—Mo4—O11	102.3 (10)	O21B—P1—O19B	109.0 (12)
O6—Mo4—O11	89.4 (7)	O21A^i^—P1—O19B^i^	69.5 (12)
O8—Mo4—O5	100.3 (9)	O21A—P1—O19B^i^	110.5 (12)
O6—Mo4—O5	89.5 (7)	O21B^i^—P1—O19B^i^	109.0 (12)
O11—Mo4—O5	157.0 (10)	O21B—P1—O19B^i^	71.0 (12)
O8—Mo4—O1	100.6 (8)	O19B—P1—O19B^i^	180 (3)
O6—Mo4—O1	156.4 (9)	O21A^i^—P1—O19A^i^	110.1 (12)
O11—Mo4—O1	85.8 (7)	O21A—P1—O19A^i^	69.9 (12)
O5—Mo4—O1	86.1 (6)	O21B^i^—P1—O19A^i^	72.6 (13)
O8—Mo4—O21B	161.3 (9)	O21B—P1—O19A^i^	107.4 (13)
O6—Mo4—O21B	65.4 (8)	O19B—P1—O19A^i^	107.2 (12)
O11—Mo4—O21B	64.4 (9)	O19B^i^—P1—O19A^i^	72.8 (12)
O5—Mo4—O21B	94.5 (8)	O21A^i^—P1—O19A	69.9 (12)
O1—Mo4—O21B	91.8 (8)	O21A—P1—O19A	110.1 (12)
O8—Mo4—O21A	159.1 (8)	O21B^i^—P1—O19A	107.4 (13)
O6—Mo4—O21A	92.4 (9)	O21B—P1—O19A	72.6 (13)
O11—Mo4—O21A	91.8 (9)	O19B—P1—O19A	72.8 (12)
O5—Mo4—O21A	65.3 (8)	O19B^i^—P1—O19A	107.2 (12)
O1—Mo4—O21A	64.7 (7)	O19A^i^—P1—O19A	180 (2)
O21B—Mo4—O21A	39.6 (8)		

Symmetry codes: (i) −*x*+1/2, −*y*+1/2, −*z*.

### Hydrogen-bond geometry (Å, °)

**Table:**

*D*—H···*A*	*D*—H	H···*A*	*D*···*A*	*D*—H···*A*
N3—H3A···O17^ii^	0.86	2.03	2.82 (3)	153
N6—H6···O2W	0.86	1.98	2.82 (5)	167
N9—H9A···O1W	0.86	1.95	2.77 (3)	157

Symmetry codes: (ii) −*x*+1/2, *y*+1/2, −*z*+1/2.

## References

[bb1] Artero, V. & Proust, A. (2000). *Eur. J. Inorg. Chem.* pp. 2393–2400

[bb2] Bruker (2001). *SAINT-Plus* and *SADABS* Bruker AXS Inc., Madison, Wisconsin, USA.

[bb3] Bruker (2004). *APEX2* Bruker AXS Inc., Madison, Wisconsin, USA.

[bb4] Hao, L., Ma, C., Chen, J., Zhang, X. & Zhang, X. (2010). *Acta Cryst.* E**66**, m231–m232. 10.1107/S160053681000320X PMC2979847 21579687

[bb5] Hao, L., Wang, Y., Zhang, X., Chen, J. & Zhang, X. (2010). *Acta Cryst.* E**66**, m268–m269. 10.1107/S1600536810004307 PMC2983543 21580219

[bb6] Kurmoo, M., Bonamico, M., Bellitto, C., Fares, V., Federici, F., Guionneau, P., Ducasse, L., Kitagawa, H. & Day, P. (1998). *Adv. Mater.* **7**, 545–550.

[bb7] Niu, J. Y., Shan, B. Z. & You, X. Z. (1999). *Transition Met. Chem.* **24**, 108–114.

[bb8] Pope, M. T. & Müller, A. (1991). *Angew. Chem. Int. Ed.* **30**, 34–38.

[bb9] Sheldrick, G. M. (2008). *Acta Cryst.* A**64**, 112–122. 10.1107/S0108767307043930 18156677

[bb10] Yang, W. B., Lu, C. Z., Wu, C. D. & Zhuang, H. H. (2003). *Chin. J. Struct. Chem.* **22**, 137–142.

[bb11] Zhang, X. T., Dou, J. M., Wei, P. H., Li, D. C., Li, B., Shi, C. W. & Hu, B. (2009). *Inorg. Chim. Acta*, **362**, 3325–3332.

[bb12] Zhang, X. T., Wei, P. H., Sun, D. F., Ni, Z. H., Dou, J. M., Li, B., Shi, C. W. & Hu, B. (2009). *Cryst. Growth Des.* **9**, 4424–4428.

